# Morphology, morphometry, and phylogeny of the protozoan parasite, *Eimeria labbeana*-like (Apicomplexa, Eimeriidae), infecting *Columba livia domestica*

**DOI:** 10.3389/fvets.2024.1392238

**Published:** 2024-05-30

**Authors:** Shurug Albasyouni, Rewaida Abdel-Gaber, Saleh Al Quraishy, Esam M. Al-Shaebi, Osama B. Mohammed

**Affiliations:** Department of Zoology, College of Science, King Saud University, Riyadh, Saudi Arabia

**Keywords:** pigeons, coccidia, molecular technique, phylogeny, Saudi Arabia

## Abstract

**Introduction:**

*Eimeria* spp. are intracellular protozoan parasites of the phylum Apicomplexa causing economic losses to various wild and domestic animals. An eimerian species infecting *Columba livia domestica* was identified in this study.

**Methods:**

A total of 15 faecal samples were examined by floatation technique, a prevalence rate of 60% was reported. Eimerian oocysts were sporulated in 2.5% potassium dichromate solution then identified using morphological and molecular (DNA amplification of the *18S rRNA* and *ITS-1* genes) diagnostic techniques.

**Results:**

Sporulated oocysts were identified as *Eimeria labbeana*-like, after morphometry with typical bi-layered wall with spherical to subspherical oocysts morphology. A polar granule is present, but no micropyle or oocyst residuum. Sporocysts are elongated ovoidal with stieda body. Sporocyst residuum with many granules and sporozoites with refractile bodies and nucleus. Both *18S rRNA* and *ITS-1* sequences have been deposited in GenBank database. DNA sequences from the partial *18S rRNA* generated from the oocysts were found to be related to eimerian and isosporan parasites found in domestic pigeons. For the first time, *ITS-1* sequences for *E. labbeana*-like were provided.

**Conclusion:**

The necessity of using molecular techniques to describe pigeon intestinal coccidian parasites in conjunction with traditional morphology-based tools was emphasized in this work in order to understand the biology of such parasites.

## Introduction

Coccidiosis is a parasitic disease of all bird’s intestinal tract caused by protistan parasites the genera of *Eimeria*, *Isospora, Caryospora*, and *Tyzzeria* (1, 2). Because of the walls of oocysts, these coccidian organisms may survive in the environment. Infected birds discharge microscopic oocysts in their feces, causing other birds to become infected via ingesting sporulated oocysts. The discharged oocysts require a time, in the surrounding environment outside the host, to sporulate to produce sporulated oocyst containing sporozoites within sporocysts (infective stage) that can infect another host, hence completing the life cycle (3). Disease may have a negative impact on farm animals by costs for treatment, prevention, eradication, decontamination, and restocking. In birds, life cycle of members of the genus *Eimeria* begins when sporulated oocysts are ingested by susceptible birds. Coccidia infiltrates the intestinal lining after being ingested, undergo both sexual and asexual reproduction, and cause tissue damage (4). Post-mortem examination of the host and fecal examination can confirm the existence of this disease (5–7).

Several species of the genus *Eimeria* have been described infecting pigeons employing the traditional morphological description, parasite biology, and typical macroscopic lesions, including *E. chalcoptereae* (8), *E. choudari* (9), *E. columbae* (10), *E. columbapalumbi* (11), *E. columbarum* (12), *E. columbinae* (13), *E. curvata* (14), *E. duculai* (15), *E. gourai* (15), *E. janovyi* (16), *E. kapotei* (17), *E. labbeana* (18), *E. labbeana-*like (19), *E. livialis* (20), *E. mauritiensis* (21), *E. palumbi* (22), *E. sphenocerae* (23), *E. tropicalis* (24), *E. turturi* (25), *E. waiganiensis* (26), and *E. zenaidae* (27). *E. labbeana* is the most pathogenic and often reported species, located in small intestine of pigeons and causing diarrhea, enteritis, and even mortality (19).

However, due to inadequate description and lack of measurements for several eimerian species from the Columbidae in the past, it has been difficult to assign and confirm identities of existing species. Duszynski et al. (28) stated that just two species (*E. labbeana* and *E. columbarum*) are likely to occur in pigeons and considered as valid species. Due to these challenges, molecular methods are required to reliably delimit taxa and infer phylogenetic relationships among members of the genus *Eimeria* (29). Several approaches based on the polymerase chain reaction (PCR) have been developed to characterize avian eimerian species, including the amplification of the nuclear genes such as small subunit (8, 13, 19, 30), large subunit (8, 19) rRNA; and the internal transcribed spacer region 1 (ITS-1) (5), as well as the mitochondrial cytochrome c oxidase subunit I (COI) (8, 13, 19, 31).

This study was carried out to describe and characterize the eimerian oocysts recovered from domestic pigeons using morphological and molecular tools.

## Materials and methods

### Sample collection

A commercial poultry farm in Riyadh (Saudi Arabia) yielded 15 specimens of domestic pigeon, (*C. livia domestica*). Pigeons were housed indoors in well-ventilated cages with free access to food and water *ad libitum* and were raised following the institution’s criteria for animal care and use in research (approval number KSU-SU-23-45).

### Fecal examination

Fecal samples, from each bird, weighing around 1 g were collected in separate screw-capped plastic containers labeled properly and delivered to the Parasitology Laboratory Research at the Department of Zoology, College of Science. The samples were initially analyzed to determine their consistency and color, as well as the presence of mucus, blood, and other contaminants. Standard microscopical procedures were used to examine the presence or absence of coccidia oocysts. Flotation technique with Sheather’s sucrose solution (specific gravity 1.27) was employed in order to concentrate the oocysts in positive samples (32).

### Sporulation of oocysts

According to Levine (33), the oocysts were placed in a 2.5% (w/v) potassium dichromate solution, left at room temperature, and checked to track the sporulation process. For further investigation, the sporulated oocysts were washed three times in phosphate-buffered saline and stored at 4°C.

### Morphology and morphometry

Following the standards of Silva et al. (2) and Saikia et al. (5), eimerian species were identified based on oocyst morphology and sporulation time. Photographs were taken with a Leica DM 2500 microscope (NIS ELEMENTS software, version 3.8). The size (including length and width) and shape index (length/width ratio) of 50 oocysts from each fecal sample were measured using ocular micrometer. All measurements are given in microns (μm) and a range (mean in parentheses) using ImageJ 1.53e software (Wayne Rasband and contributors, National Institute of Health, United States).

### Molecular techniques

#### DNA extraction

Purified oocysts were suspended in 100 μL sodium hypochlorite at 65°C for 45 min. For 1 h at 65°C, the samples were combined with 350 μL of CTAB extraction buffer (2% cetyltrimethylammonium bromide, 1% polyvinylpyrrolidone, 100 mM Tris–HCl, 1.4 M NaCl, 20 mM EDTA) (34). An ultrasonicator (Thermo Fischer Scientific, United States) was used to disrupt the rigid wall of sporulated oocysts. The genomic DNA was extracted from excysted sporozoites using Isolate II fecal DNA extraction kit (Meridian Bioscience, London, United Kingdom). DNA samples were kept at −20°C until further processing.

#### Polymerase chain reaction

The methods described by Al-Quraishy et al. (35) to amplify the *18S rRNA* and *ITS-1* regions were used for PCR. The PCR reaction was carried out in accordance with the suggested PCR conditions and the genus-specific primers published by Orlandi et al. (36) for the *18S rRNA* and Kawahara et al. (37) for the *ITS-1* regions. Gel electrophoresis of amplified DNA was run on 1.5% (w/v) agarose gel (Sigma-Aldrich, United States) stained with SYBR Safe DNA gel dye (Thermo Fischer Scientific, Canada) was used to visualize PCR results. The gel was loaded with a DNA ladder (100 bp DNA, Fermentas) and the expected product size was visualized using a gel documentation system (BioRad, United States).

#### Sequencing and phylogenetic analysis

Positive PCR products were sequenced in the forward direction using Macrogen^®^ sequencing facility (Seoul, South Korea). The identity of the generated sequences was checked using a BLAST search and aligned with relevant sequences using the CLUSTAL-X method (38). The phylogenetic trees were generated using Bayesian Inference (BI) and maximum likelihood (ML) methods using Mr. Bayes and MEGA 11 software, respectively (39, 40). Distances were estimated using the Kimura 2-parameter model, and the numbers at the branch of the tree demonstrate bootstrap support from 1,000 replications. Markov Chain Monte Carlo chains were run for 2,000,000 generations, the log-likelihood scores were plotted, and the final 75% of trees were used to produce consensus trees. The *18S rRNA* gene sequence of *Toxoplasma gondii* (L24381) was included in the tree as an outgroup.

## Results

Gross examination revealed color and consistency variations in the fecal samples, including greenish feces and watery diarrhea, in 9 of 15 samples. Microscopic examination recorded that that 60% (*n* = 9) of 15 fecal samples contained unsporulated coccidian oocysts, and the affected pigeons expressed weakness and reduced appetite. Unsporulated oocysts reached full sporulation after 1–2 days when left at 2.5% K_2_Cr_2_O_7_ at room temperature (25± 2°C). Sporulated oocysts recovered in the present study correspond with the description criteria of the genus *Eimeria*, with close similarity to *Eimeria labbeana*-like as described below.

### Morphology and morphometry

**Figure 1:**
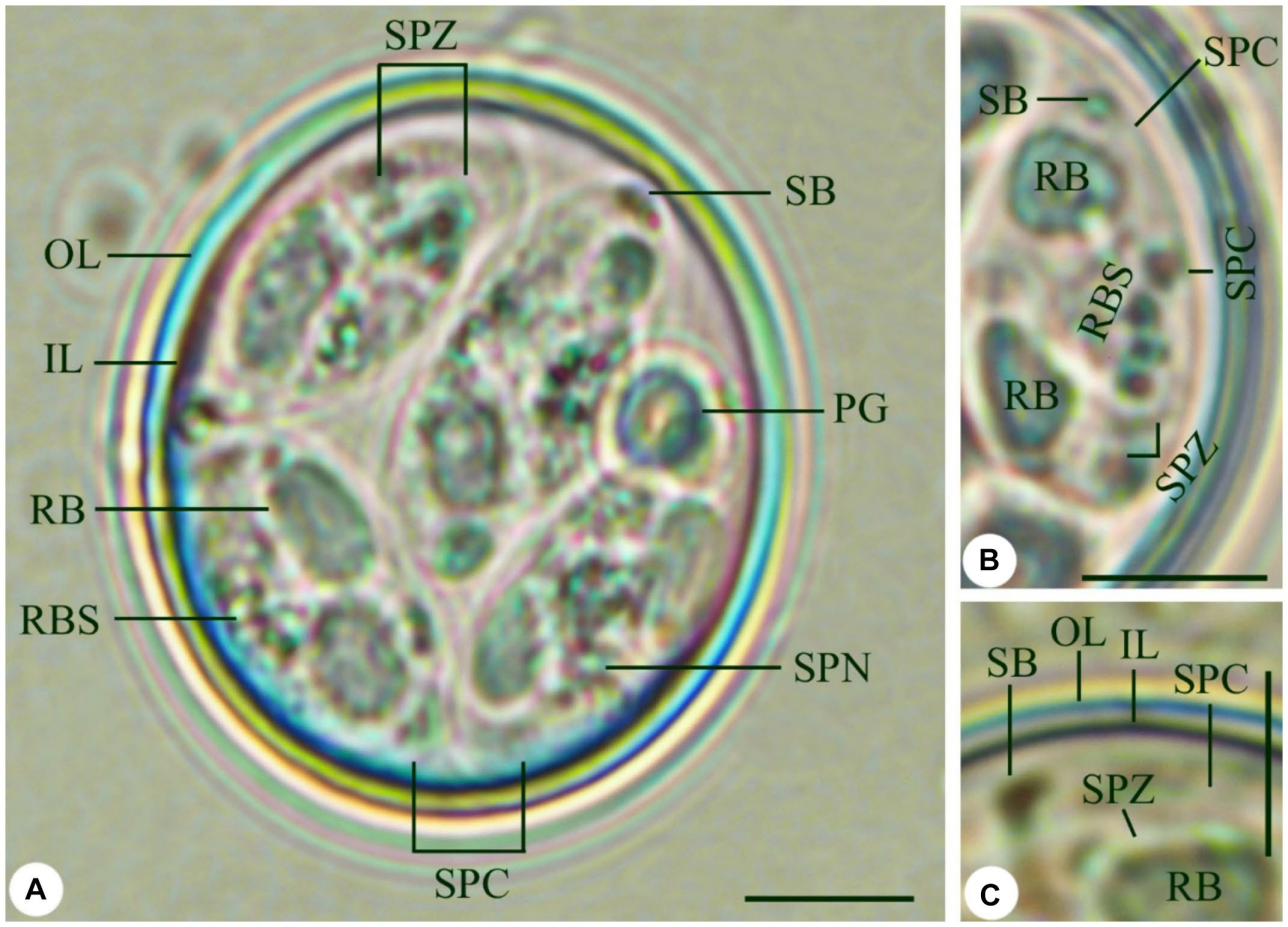
*Eimeria labbeana*-like infecting pigeons. **(A)** Sporulated oocyst. **(B,C)** High magnifications of sporocyst with sporozoites and refractile body (OL, Outer layer; IL, Inner layer; RF, Refractile body; SB, Stieda body; PG, Polar granule; SPC, Sporocyst; RBS, Residuum of sporocyst; SPZ, Sporozoite; SPN, Sporozoite nucleus) Scale = 5 μm.

The sporulated oocysts were spherical to subspherical in shape (Figure 1A). The oocyst wall was bilayered (Figures 1A,C), the outer layer was thinner than the inner layer measuring 1.4–1.7 (1.5). Fifty oocysts were measured, with sizes ranging from 18.8 to 21.9 in length and 15.9–16.7 in width (Table 1). The average size was 20.4 × 16.4 μm without a micropyle or oocyst residuum (Table 1). Their length-width ratio (shape index) ranged from 1.2 to 1.3 (1.2) (Table 1). The oocyst possessed an ovoid polar granule. Oocysts sporulation within 24–36 h. The sporocysts were elongated ovoidal with a single-layered (Figures 1A,B), ranging in size from 11.9 to 13.8 in length and 5.1–6.5 in width (Table 1). Sporocysts had an average size of 12.7 × 5.9 μm (Table 1). Their shape index ranged from 1.9 to 2.1 with a mean of 2.1. Stieda body was present, 0.7–1.0 (0.8) × 1.2–0.9 (1.1) μm, however, substieda body is not present. A sporocyst residuum is a spherical mass made up of several granules (Figures 1A,B). Sporozoites were elongated, lying lengthwise head to tail inside the sporocyst, with two refractile bodies (Figures 1A–C), one of which is spherical and 3.1–3.8 (3.5) × 1.5–2.2 (1.9) μm. A nucleus was seen directly in the posterior refractile body (Figures 1A,B).

**Table 1:** Morphological characteristics of sporulated oocysts for the recovered *Eimeria labbeana* and *E. labbeana-*like species from Columbidae.

*Eimeria* species	Host species	Oocysts			Micropyle	Residuum	Sporocyst size		Locality
		Shape	Length	Width			Length	Width	
*Eimeria labbeana* Pinto (18)	*C. livia*	Subspherical to ovoidal	17–21	16–18	+	−	11–14 (12.4)	5–7 (6.4)	Asia, India
*Eimeria labbeana* Nieschulz (12)	*C. livia*	Subspherical to ellipsoidal	15–18 (16.7)	14–16 (15.3)	−	−	12.4	6.4	Asia, India
*Eimeria labbeana*-like Yang et al. (19)	*C. livia*	Subspherical	18.9–22 (20.2)	15.7–18.9 (16.1)	−	+	12.5–14.5 (13)	5.5–7 (6.1)	Australia
*Eimeria labbeana* Elseify et al. (45)	*Coturnix ypsilophora*	Subspherical to spherical	21.5–22.6	16.9–19.8	−	– –	10.54–16.68	6.2–10.6	Egypt
*Eimeria labbeana* Saikia et al. (5)	*C. livia domestica*	Subspherical to spherical	19.50–23.43 (21.02)	16.41–19.03 (17.98)	−	−	– –	– –	India
*Eimeria labbeana* Joseph et al. (46)	*C. livia domestica*	Subspherical	16.5	15	−	−	– –	– –	Nigeria
*Eimeria labbeana* Aboelhadid et al. (52)	*C. livia domestica*	Subspherical to ovoidal	15–18.9	14–17.5	−	−	– –	– –	Egypt
*Eimeria labbeana* Al-Agouri et al. (53)	*C. livia domestica*	Subspherical to spherical	16.5	15	−	+	– –	– –	Libya
*Eimeria labbeana* Oliveira et al. (30)	*Streptopelia decaocto*	Subspherical to ellipsoidal	16–21 (18.7)	14–18 (15.7)	+	−	10–14 (12.2)	5–7 (6.4)	Portugal
	*C. palumbus*	Subspherical to ellipsoidal	16–21 (19)	14–18 (15.9)	+	−	10–14 (12.3)	5–7 (6.0)	Portugal
*Eimeria labbeana-*like(Present study)	*C. livia domestica*	Subspherical to spherical	18.8–21.9 (20.4)	15.9–16.7 (16.4)	−	−	11.9–13.8 (12.7)	5.1–6.5 (5.9)	Saudi Arabia

+ present, − absent, – – not detected.

### Molecular analysis

**Figure 2:**
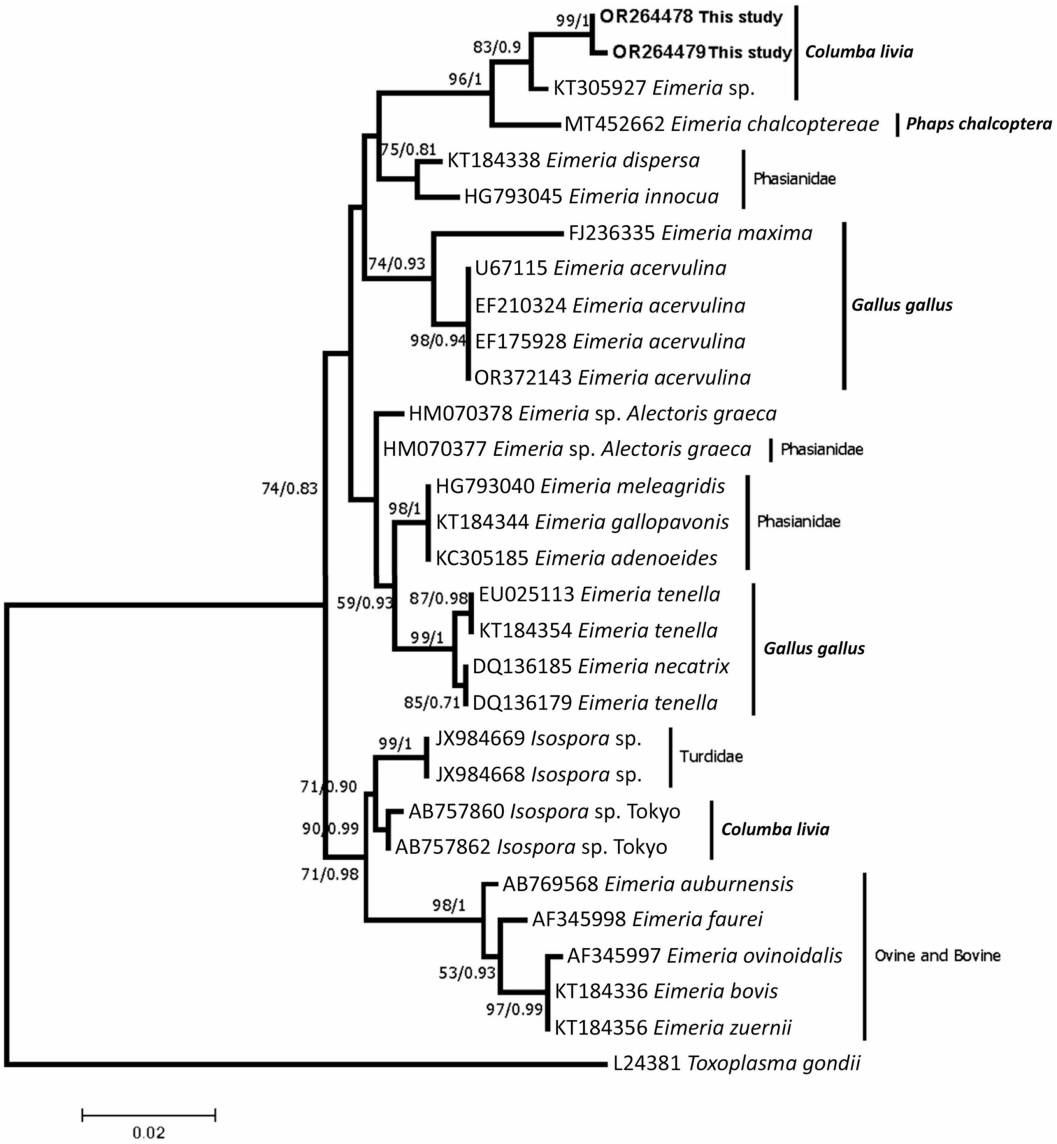
A consensus phylogenetic tree constructed with maximum likelihood (ML) and Bayesian Inference (BI) methods, showing phylogenetic relationships between *Eimeria labbeana*-like and related taxa in NCBI GenBank database with *Toxoplasma gondii* as an outgroup. The ML and BI trees are inferred from the partial *18S rRNA* sequences data generated from the *E. labbeana*-like detected from *C. livia domestica* (OR264478 and OR264479 shown in bold) and related taxa from GenBank database. Numbers indicated at branch nodes are bootstrap values and posterior probability (ML/BI). Only bootstraps >50% are shown.

Partial *18S rRNA* and *ITS-1* gene regions were successfully amplified and yielded ~613 and ~ 600 bp, respectively. Two sequences of were obtained from the partial *18S rRNA* and were deposited in GenBank database with the accession numbers OR264478 and OR264479. The two sequences were identical with only one mutation at position 182 of the alignment (with a transversion C/G). Phylogenetic analysis revealed that the two sequences generated from the *E. labbeana*-like in the present study shared a common ancestor with *E. labbeana*-like from the GenBank database (KT305927 from *C. livia domestica* from Australia) with high ML bootstrap values and high BI posterior probability as shown in Figure 2. Furthermore, they clustered with DNA sequences of the same region obtained from *Eimeria* spp. from Columbidae. They were distinct from those *Eimeria* spp. from Phasianidae and Turdidae. Three Sequences were obtained from the *ITS-1* region and were deposited at GenBank database with the accession numbers OR270024-OR270026. The obtained sequences were different from all *ITS-1* sequences deposited in GenBank database with an identity of less than 75%. However, the last part of the sequences (80 bp) which constitutes the *5.8S rRNA* region was highly similar to several eimerian species with 100% identity to *E. subspherica* of bovines.

## Discussion

The fecal examination is the most commonly used laboratory technique in veterinary practice for diagnosis of the parasitic infections (41). According to the current data, multiple methods for identification of *Eimeria* species are utilized in field diagnosis. In the current study, 9 of 15 samples tested positive for coccidian oocysts, yielding an overall prevalence of 60% which is in agreement with different reports from various countries (75% by Ramesh et al. (42) from Chennai (India), 67.58% by Gül et al. (43) from Van City (Turkey), 61.36% by El-Sayed (44) from Sharkia Governorate (Egypt), 59.6% by Aleksandra and Pilarczyk (6) from Pomerania province (German), 58.2% by Elseify et al. (45) from Qena province (Egypt), 56.2% by Joseph et al. (46) from Maiduguri Metropolis Borno State (Nigeria), and 52% by Hui et al. (47) from Shanghai (China)). It has been reported that young and growing pigeons lack acquired immunity to coccidian infections and outbreaks can occur under conditions of poor hygiene. Clinical manifestation of pigeon intestinal coccidiosis appeared in the form of greenish watery diarrhea, a decrease in food intake, and body weakness. These findings are consistent with those published by Bandyopadhyay et al. (16), Dalloul and Lillehoj (48), Bhrami et al. (49), Quiroz-Castañeda et al. (50), and Gadelhaq and Abdelaty (51), who all found that coccidiosis had pathological effects on domestic pigeons, resulting in significant losses.

Researchers used different criteria to identify eimerian species excreted in the droppings of pigeons including the morphology and morphometry of oocysts, pre- and patent periods, and sporulation time (5, 11, 13, 19, 29, 51, 52). Based on morphology, it has been confirmed that *E. labbeana*-like is infecting pigeons in a commercial poultry farm in Riyadh area (Saudi Arabia). Yang et al. (19) found oocysts with similar morphological features from coccidian infection in *C. livia* in Australia, however, they have reported oocysts with oocystic residuum, which is not visible in their photomicrographs and may corresponded to some debris stuck externally to the oocyst wall. When comparing the oocysts detected in the present study with the group of *E. labbeana* species previously described from the Columbidae, the following findings can be made: (i) The oocyst studied in this study, or those from Australia, was far from the type locality of *E. labbeana*. (ii) The morphometric data of the oocysts showed variation in the size of the oocysts which were larger than that described by Nieschulz (12), Joseph et al. (46), Aboelhadid et al. (52), Al-Agouri et al. (53), and Oliveira et al. (30). (iii) The oocyst shape of *E. labbeana* was spherical to subspherical except for those described by Pinto (18), Nieschulz (12), Aboelhadid et al. (52), and Oliveira et al. (30) who highlighted the polymorphic nature of the oocysts, which could be sub-spherical and/or ellipsoidal. (iv) There was no micropyle except for those identified by Pinto (18) and Oliveira et al. (30). (v) There was no oocyst residuum except for those described by Al-Agouri et al. (53).

Partial *18S rRNA* sequences of the eimerian oocysts from the present study indicated that the sequences are related to the 18S rDNA sequences obtained from eimerian parasites from the Columbidae. One of the sequences (KT305927) obtained from *Eimeria* sp. which regarded by Yang et al. (8) as *E. labbeana*-like from *C. l. domestica* in Australia. However, three sequences from *Isospora* sp. (AB757861, AB757863, AB757864) obtained from *C. l. domestica* from Japan and a sequence from *E. chalcoptereae* from a bronzewing pigeon (*Phaps chalcoptera*) in Australia (8). The *18S rRNA* sequences obtained in the present study differed from those from *Isospora* sp. and *E. chalcoptereae*, However, they showed high similarity to sequences from *E. labbeana*-like reported by Yang et al. (19) with 98.5% similarity. Morphological description of *E. labbeana* or *E. labbeana*-like oocysts showed remarkable variation. Since molecular data for *E. labbeana*-like were only available from Yang et al. (19) and the present study. We, therefore, suggest that the sequences reported in the present study and that reported by Yang et al. (19), since they have a high similarity of 98.5%, may probably be for the same species which was *E. labbeana*-like. Even though they were from two different and distant localities and they were similar in morphology and morphometry except for the presence of oocyst residuum in the oocysts of Yang et al. (19). All other descriptions of *E. labbeana* did not show oocyst residuum except for those descriptions from Yang et al. (19) and Al-Agouri et al. (53). Both Yang et al. (19) and Al-Agouri et al. (53) in their description of *E. labbeana*-like or *E. labbeana* mentioned the presence of oocyst residuum, however, the oocyst residuum was inconspicuous in their photographs which may probably be an artifact. During the present study, we have reported sequences for the *ITS-1* and the 5.8S rRNA regions and there were no sequences for *E. labbeana* or related *Eimeria* spp. which found in GenBank database. Yang et al. (19) studied the cytochrome c oxidase I sequence variation in *E. labbeana*-like and they found it related to *E. dispersa* from the wild turkey (*Meleagris gallopavo*). This probably resulted from the unavailability of related sequences in GenBank database. Despite repeated attempts, it was not possible to obtain sequences from cytochrome c oxidase I in the present study.

## Conclusion

This study provides additional knowledge about the oocysts of *Eimeria labbeana*-like in *C. livia domestica* (its type host) from Riyadh (Saudi Arabia). Moreover, unique genetic sequences were added in GenBank database for *18S rRNA* and *ITS-1* regions that recovered for this eimerian species. More research is needed to incorporate preventative and control approaches to reduce the economic impact of *E. labbeana*-like infection.

## Data availability statement

The data presented in the study are deposited in the parasitological collection of the museum, College of Science, King Saud University, Riyadh, Saudi Arabia. Two DNA sequences of partial 18S rRNA gene were deposited at GenBank and were given the accession numbers OR264478 and OR264479. In addition to three additional sequence of partial *ITS-1* gene region with the accession numbers OR270024-OR270026.

## Ethics statement

The animal study was approved by the Research Ethical Committee (REC) at King Saud University. The study was conducted in accordance with the local legislation and institutional requirements.

## Author contributions

SA: Methodology, Resources, Software, Writing – review & editing, Conceptualization, Data curation, Investigation, Project administration, Supervision, Validation, Visualization, Writing – original draft, Formal analysis. RA-G: Conceptualization, Data curation, Formal analysis, Funding acquisition, Investigation, Methodology, Project administration, Resources, Software, Supervision, Validation, Visualization, Writing – original draft, Writing – review & editing. SAQ: Project administration, Resources, Software, Writing – original draft, Data curation, Investigation, Supervision, Validation, Visualization, Writing – review & editing, Conceptualization, Formal analysis, Methodology. EA-S: Formal analysis, Methodology, Resources, Software, Visualization, Writing – review & editing, Conceptualization, Data curation, Investigation, Project administration, Supervision, Validation, Writing – original draft. OM: Conceptualization, Formal analysis, Methodology, Visualization, Writing – original draft, Writing – review & editing, Data curation, Investigation, Project administration, Resources, Software, Supervision, Validation.
